# Inflammatory but not apoptotic death of granulocytes citrullinates fibrinogen

**DOI:** 10.1186/s13075-015-0890-0

**Published:** 2015-12-17

**Authors:** Nathalie E. Blachère, Salina Parveen, John Fak, Mayu O. Frank, Dana E. Orange

**Affiliations:** Laboratory of Neuro-Oncology, The Rockefeller University, 1230 York Avenue, New York, NY 10065 USA; Howard Hughes Medical Institute, New York, New York 10065 USA; Division of Rheumatology, Hospital for Special Surgery, New York, NY 10021 USA; New York Genome Center, 101 Avenue of the Americas, New York, NY 10013 USA

**Keywords:** Rheumatoid arthritis, Citrullination, Fibrinogen, Inflammation, NETosis, Necrosis, Apoptosis, ACPA, Neutrophils

## Abstract

**Background:**

Neutrophil activation induces citrullination of intracellular targets of anticitrullinated peptide antibodies (ACPA), which are specific for rheumatoid arthritis (RA). Citrullinated fibrinogen is bound by ACPA but it is less well understood how extracellular proteins are citrullinated. The cells that produce fibrinogen, hepatocytes, do not express peptidyl arginine deiminase (PAD) enzymes nor do PAD enzymes include N-terminal signal peptides to direct them into the secretory pathway. We hypothesized that dying neutrophils release PAD in the extracellular space, and that this could cause citrullination of target extracellular antigens relevant to RA such as fibrinogen.

**Methods:**

HL60 cells were differentiated into neutrophil-like cells by treatment with all-*trans* retinoic acid (ATRA). Differentiation was confirmed by CD11b staining, PAD4, PAD2 and myeloperoxidase expression, cell division, and nuclear morphology. Death was induced with various stimuli, including freeze-thaw to induce necrosis, Ionomycin and PMA to induce NETosis, and UV-B to induce apoptosis. Death markers were assessed by immunohistochemistry and flow cytometry. To quantify extracellular citrullination, dying ATRA-differentiated HL60 cells were cultured with fibrinogen for 24 hours and supernatants were probed for fibrinogen citrullination, PAD2 and PAD4 by western blot.

**Results:**

While both NETotic and necrotic ATRA differentiated HL60 cells citrullinated fibrinogen, apoptotic cells did not citrullinate fibrinogen, even when allowed to undergo secondary necrosis. Incubation of necrotic neutrophil lysates with fibrinogen also causes fibrinogen citrullination. PAD2 and PAD4 were detected by western blot of supernatants of ATRA-differentiated HL60 cells undergoing necrotic and NETotic death, but not apoptotic or secondarily necrotic cell death.

**Conclusion:**

We implicate granulocytes undergoing inflammatory cell death as a mechanism for altering extracellular self-proteins that may be targets of autoimmunity linked to inflammatory diseases such as rheumatoid arthritis.

**Electronic supplementary material:**

The online version of this article (doi:10.1186/s13075-015-0890-0) contains supplementary material, which is available to authorized users.

## Background

The majority of patients with rheumatoid arthritis (RA) harbor anticitrullinated peptide antibodies (ACPA), which are markers of disease severity. ACPA bind citrullinated isoforms of numerous intracellular antigens including vimentin and alpha enolase. Recent work has identified hypercitrullination of multiple intracellular neutrophil antigens after exposure to pore forming stimuli such as Ionomycin, complement and granzyme B [1] and on neutrophil extracellular traps (NETs) [2].

In addition to targeting intracellular citrullinated antigens, ACPA also bind citrullinated isoforms of various extracellular antigens. One such citrullinated extracellular antigen recognized by ACPA is citrullinated fibrinogen. Autoantibodies to citrullinated fibrinogen are 98 % specific for RA [3] and proteomic studies of synovial tissue and fluid have identified citrullinated fibrinogen as one of the major sources of citrullinated antigen in the joints in RA [4–6]. It is not known how extracellular proteins such as fibrinogen are citrullinated because the cells that produce fibrinogen, hepatocytes, do not express any of the peptidylarginine deiminase (PAD) family of enzymes nor do any of the PAD enzymes include N-terminal signal peptides that would direct them into the secretory pathway [7]. PAD4 is required for histone 3 (H3) citrullination and NETosis and recent work has shown that PAD4 may also be extruded on NETs [8]. Synovial fluid from patients with RA citrullinates fibrinogen in vitro, suggesting PAD enzymes in RA are externalized in the synovial fluid [9]. Though both PAD2 and PAD4 citrullinate fibrinogen [9], it remains unclear under which conditions PAD enzymes function in the extracellular compartment and whether PAD4 enmeshed in NET DNA retains enzymatic activity. A better understanding of the mechanisms by which fibrinogen can become citrullinated in the context of inflammation would facilitate dissection of the immune pathways that lead to autoreactivity to citrullinated extracellular antigens.

We hypothesized that dying neutrophils release PAD enzymes that remain functional in the extracellular compartment, and that this release could cause citrullination of target antigens relevant to RA such as fibrinogen. We therefore compared citrullination of fibrinogen cultured with granulocytes undergoing various types of cell death and discovered that apoptotic cells do not citrullinate fibrinogen, even when allowed to undergo secondary necrosis, but both NETotic and necrotic granulocytes citrullinate fibrinogen in culture. We therefore implicate neutrophils undergoing inflammatory cell death as a mechanism for altering extracellular self-proteins that may be targets of autoimmunity linked to inflammatory diseases such as RA.

## Methods

### Differentiating HL60 cells

HL60 cells were obtained from ATCC (Manassas, VA #CCL-240) and maintained in RPMI 1640 media (Invitrogen, Grand Island, NY, USA) supplemented with 20 % fetal bovine serum (FBS), 2 mM L-glutamine, 25 mM hydoxyethyl piperazineethanesulfonic acid (HEPES, Invitrogen), non-essential amino acid (Invitrogen), sodium pyruvate (Cellgro, Manassas, VA, USA) and gentamycin (Invitrogen). Cells were maintained at a density of 1 × 10^5^ to 5 × 10^5^ cells/ml for a maximum of 30 passages. Cells were treated with varying concentrations of all-trans retinoic acid (ATRA) (Sigma, St. Louis, MO, USA) dissolved in dimethyl sulfoxide (DMSO) (Sigma-Aldrich) for varying amounts of time as described in the text.

### RT-qPCR

mRNA was isolated from HL60 and ATRA HL60 with Trizol (Invitrogen) and High Pure RNA Isolation kit (Roche Diagnostics, Mannheim Germany), cDNA was synthesized with iScript (BioRad, Hercules, CA, USA). Primer pair sequences were: PADI4 forward: GCACAACATGGACTTCTACGTGG, reverse: CACGCTGTCTTGGAACACCACA; HRP14 forward: CGGAGCTGACCAGACTTTTC, reverse: GGTTCGACCGTCATACTTCTTC. MPO forward: GAGCAGGACAAATACCGCACCA, reverse: AGAGAAGCCGTCCTCATACTCC; PADI2 forward: GATGAGCAGCAAGCGAATCACC, reverse: GCTCCTTCTTGAGGATGTCACG. Specificity (melting-curve analysis) and priming efficiency (standard curve) was confirmed. BioRad CFX96 system and FastStart SYBR Green Master (Roche) were used for real-time PCR.

### Microscopy

Cells were seeded onto polylysine-D coated culture slides (Corning, Tewksbury, MA, USA), incubated at 37 °C for 30 minutes, fixed with 4 % paraformaldehyde at room temperature for 15 minutes, and permeablized with PBS containing 0.1 % Triton-X100 (Sigma-Aldrich). Cells were stained with 4,6-diamidino-2-phenylindole (DAPI) (Sigma-Aldrich) and images were obtained with an Axioplan 2 microscope (Zeiss, Oberkochen, Germany).

### Flow cytometry

First, 10^5^–10^6^ cells were stained with CD11b-FITC (BD Pharmingen, San Jose, CA, USA) in staining buffer (PBS with 1 % pooled human serum and 1 % FBS), washed and resuspended in AnnexinV-staining buffer (Molecular Probes, Thermo Fisher, Waltham Massachusetts) with AnnexinV-FITC (BD Pharmingen) for 15 minutes, 37 °C. Prior to analysis, cells were treated with 100 nM of cell impermeant nucleic acid stain (TOPRO-3 iodide) (Invitrogen).

### Ionomycin and PMA treatment

ATRA/HL60 were resuspended at 5 × 10^6^/ml in media (Hanks balanced salt solution (HBSS)) supplemented with 5 mM CaCl_2_, 5 mM DL-Dithiothreitol (DTT) (Sigma-Aldrich), 0.25 mM HEPES (Invitrogen) and where indicated, 1 mg/ml fibrinogen (Sigma-Aldrich) and cultured with 100 nM Ionomycin (Sigma-Aldrich) or 100 nM phorbol 12-myristate 13-acetate (PMA) (Sigma-Aldrich).

### UV irradiation

ATRA/HL60 were plated at 1 × 10^6^/ml in media (HBSS supplemented with 2–5 mM CaCl_2_, 5 mM DL-Dithiothreitol (DTT) (Sigma-Aldrich), 0.25 mM HEPES (Invitrogen) and where indicated, 1 mg/ml fibrinogen (Sigma-Aldrich), treated with 120 mJ/cm^2^ of UV-B irradiation and incubated at 37 °C for various durations.

### Freeze thaw

ATRA/HL60 were resuspended at 5 × 10^6^/ml in media (HBSS supplemented with 2–5 mM HEPES (Invitrogen) and where indicated, 1 mg/ml fibrinogen (Sigma-Aldrich) and treated with four4 cycles of alternating dry ice and quick thaw in a 37 °C water bath.

### Staurosporine treatment

ATRA/HL60 cells were resuspended at 5 × 10^6^/ml in media (HBSS supplemented with 2 mM CaCl_2_, 5 mM DTT (Sigma-Aldrich), 0.25 mM HEPES (Invitrogen) and where indicated, 100 ug of fibrinogen (Sigma-Aldrich) and treated with 1 uM staurosporine (Sigma-Aldrich).

### Western blots

ATRA/HL60 treated with various death-inducing stimuli as above, were incubated overnight at 37 °C. Supernatant was transferred to an eppendorf tube, centrifuged at 2,000 rpm for 2 minutes at room temperature, resuspended in 4 × sample buffer, boiled for 5 minutes, and centrifuged again at 14,000 rpm for 5 minutes. Then 25 ul were resolved by polyacrylamide gel electrophoresis and transferred to polyvinylidene fluoride (PVDF) membranes (Millipore). The membranes were blocked in 5 % non-fat milk in Tris-buffered saline (TBS) containing 0.1 % Tween 20, for 1 hour at room temperature. Membranes were probed with antibodies: monoclonal anti-citrullinated fibrinogen (Cayman Chemical, Ann Arbor, MI, USA), monoclonal anti-PAD2 antibody [10] (Abnova, #H00011240-M01, Taipei City, Taiwan), anti-PAD4 antibody [11] (Abcam #ab128086, Cambridge, MA, USA), monoclonal anti-fibrinogen (Abcam #ab10066), anti-citrullinated H3 (Abcam #ab5103), anti-glyceraldehyde-3-phosphate dehydrogenase (GAPDH) (Life Technologies #AM4300, Grand Island, NY, USA) and bound immunoglobulin was detected with horseradish peroxidase-linked secondary antibodies (Life Technologies). Reactivity was visualized with an ECL (enhanced chemilluminescence) substrate (Western Lightning® Plus-ECL, Perkin Elmer, Waltham Massachusetts) system.

### Peripheral blood neutrophils (PMN) western blot

In accordance with the Institutional Review Board reviewed protocol (RUH DOR0722), PMN were isolated using a Ficoll-paque plus (GE Healthcare, Pittsburgh, PA, USA) density gradient followed by hypotonic lysis. Cells (1 × 10^6^) were subjected to four cycles of freeze thaw in 200 ul of media (HBSS supplemented with 10 mM CaCl_2_, 5 mM DTT (Sigma-Aldrich), 0.25 mM HEPES (Invitrogen) and 100 ug of fibrinogen (Sigma-Aldrich) and incubated for various durations. Soluble fractions were harvested, resolved and probed as above.

## Results

### ATRA differentiates HL60 into neutrophil-like cells

**Fig. 1:**
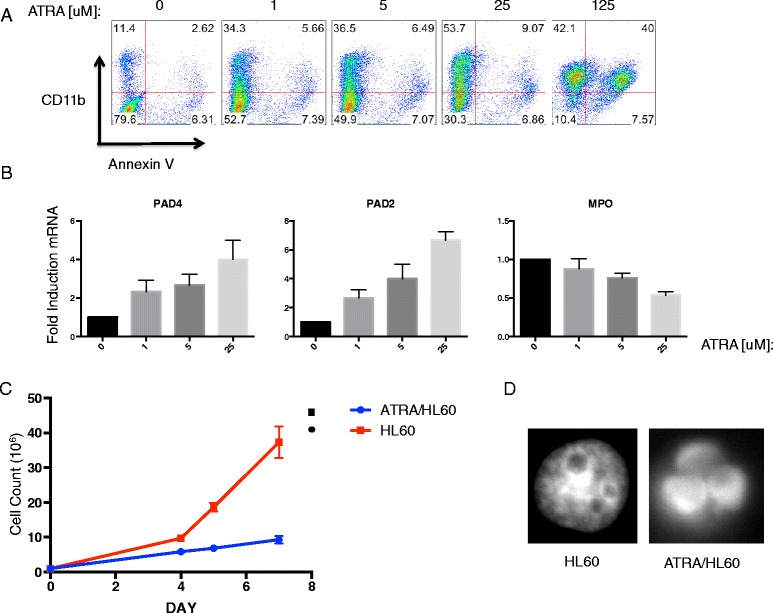
Trans retinoic acid (*ATRA*) differentiates HL60 acute promyelocytic cell line to neutrophil-like cells. **a** CD11b and Annexin V staining of HL60 cells treated with various concentrations of ATRA for 3 days. **b** qPCR of HL60 cells treated with various concentrations of ATRA for 3 days. Data are mean fold-induction of mRNA normalized to untreated HL60 cells (scored as 1) + SD of triplicate wells. **c** Growth curve of cultured HL60 cells or ATRA-treated HL60 cells over time. Cells were plated at a density of 1 × 10^5^/ml and split when the concentration exceeded 5 × 10^5^/ml. Data are mean cell counts +/− SD of triplicate culture plates (**d**) 4,6-diamidino-2-phenylindole staining of nuclear morphology of ATRA-treated or untreated HL60 cells. All data are representative of three independent experiments. *PAD* peptidylarginine deiminase

Because a significant percentage of primary neutrophils undergo spontaneous apoptosis and necrosis over time in response to basic in vitro manipulation, we used the acute promyelocytic leukemia cell line, HL60 cells, to more easily control cell death in culture. We first sought to confirm that the treatment of HL60 cells with ATRA terminally differentiates them to become neutrophil-like in culture. HL60 were treated with varying concentrations of ATRA or left untreated. Survival and CD11b expression were measured 3 days later. ATRA-treated HL60 cells upregulated CD11b in a dose-dependent manner. They become apoptotic when exposed to very high concentrations (125 uM) of ATRA (Fig. 1a). Consistent with differentiation to a more mature phenotype, ATRA-treated HL60 upregulated expression of PAD4 and PAD2 mRNA, while they downregulated expression of myeloperoxidase mRNA (Fig. 1b). Because 25 uM ATRA led to the highest CD11b expression without inducing apoptosis, this concentration was used for future experiments to induce differentiation. Growth curves confirmed that 4 days of ATRA treatment caused HL60 cells to stop dividing (Fig. 1c) and their nuclear morphology to become multi-lobar (Fig. 1d). Taken together, these data confirmed that ATRA treatment differentiated HL60 cells (ATRA/HL60) into a neutrophil-like cell, with increased expression of PAD2 and PAD4 and cell surface expression of CD11b.

### ATRA/HL60 cells treated with Ionomycin, PMA and UV-B undergo distinct forms of cell death

**Fig. 2:**
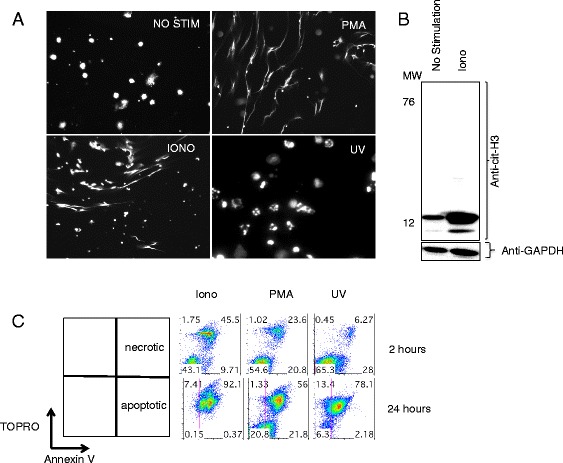
Ionomycin, phorbol 12-myristate 13-acetate (*PMA*) and UV-B induce distinct forms of granulocyte cell death. **a** Trans retinoic acid (ATRA)-differentiated HL60 cells treated with no stimulation, Ionomycin, PMA, or UV-B and stained with 4,6-diamidino-2-phenylindole. **b** Unstimulated (*NO STIM*) and Ionomycin-treated (*IONO*) granulocytes screened by western blot for citrullination of H3. Blots were stripped and reprobed for glyceraldehyde-3-phosphate dehydrogenase (*GAPDH*) as a load control. **c** Flow cytometry (ungated) of ATRA-differentiated HL60 cells treated with Ionomycin, PMA or UV-B and stained with Annexin V and cell impermeant nucleic acid stain (TOPRO). Data are representative of at least three independent experiments

We next sought to generate different types of cell death in ATRA/HL60. Cells were treated with Ionomycin, PMA or UV-B irradiation and nuclear morphology was evaluated by DAPI staining. Ionomycin and PMA-treated cells developed nuclear morphology consistent with NETosis, while UV-irradiated cells were characterized by nuclear blebs consistent with apoptosis (Fig. 2a). A limitation of assessing death by nuclear morphology is that it is difficult to definitively distinguish between NETosis and necrosis. NETosis is associated with increased citrullination of H3. Western blots of Ionomycin-treated cell lysates demonstrated increased citrullinated H3 relative to unstimulated cells (Fig. 2b), consistent with the NETotic nuclear morphology seen in Fig. 2a. PMA and Ionomycin treatment induced the same amount of citrullination of histone H3 at 2 hours (Additional file 1), but at 6 hours, the result for PMA treatment was no different to that for unstimulated cells, consistent with a prior report [12].

To better quantitate the extent of necrosis and apoptosis in the various conditions, we compared Annexin V and TOPRO labeling at several time points (Fig. 2c). Each stimulus led to a mix of various types of cell death. Ionomycin treatment led to death of 57 % of the cells in 2 hours. Ionomycin induced death was largely characterized by breakdown of cell membrane integrity (TOPRO^+^) and progressed to involve 92 % of cells at 24 hours. PMA induced death in 45 % of the cells at 2 hours, however, this was characterized by two distinct populations, TOPRO^+^ and TOPRO^–^/Annexin V^+^ cells. Interestingly, 43 % of cells still excluded TOPRO after 24 hours of PMA treatment. UV irradiation predominantly produced a population of apoptotic cells (Annexin V^+^/TOPRO^–^) at 2 hours, which largely became secondarily necrotic at 24 hours (Additional file 2). Taken together, these results demonstrate that UV irradiation induces apoptosis and ultimately secondary necrosis while Ionomycin treatment induces NETosis and necrosis and PMA treatment induces a mix of various types of cell death including NETosis and apoptosis.

### Inflammatory, but not apoptotic, granulocyte death leads to citrullination of fibrinogen

**Fig. 3:**
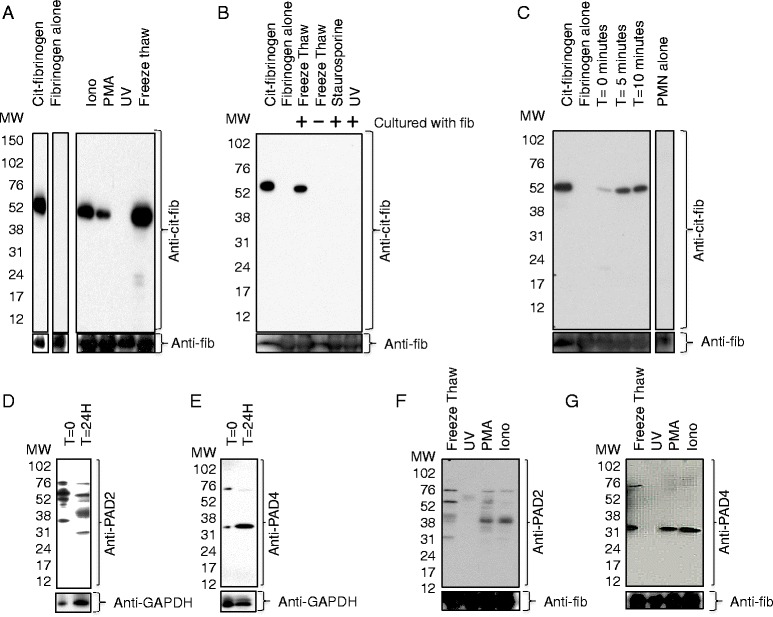
Inflammatory, but not apoptotic, granulocyte death leads to citrullination of fibrinogen. **a** Western blot of supernatants of trans retinoic acid (ATRA)/HL60 cells treated with indicated death-inducing stimuli and cultured in Hanks balanced salt solution (HBSS)/fibrinogen/5 mM calcium for 24 hours. Purified citrullinated fibrinogen and fibrinogen (without added ATRA/HL60 cells) were run as positive and negative controls, respectively. These lanes were not adjacent on the original gel. Blots were stripped and reprobed with anti-fibrinogen antibody as a load control. **b** Western blot of supernatants of ATRA/HL60 cells treated with indicated death-inducing stimuli and incubated in HBSS with or without fibrinogen and 2 mM calcium for 24 hours. **c** Western blot of primary human neutrophil lysate incubated with fibrinogen/calcium for indicated times. Peripheral blood neutrophils (*PMN*) alone (*PMN alone*) neutrophil lysate without fibrinogen, was not adjacent on the original gel. Western blots of time (T) = 0 ATRA/HL60 cell pellet or soluble fraction of ATRA/HL60 cells treated with four cycles of freeze thaw and then incubated for 24 hours and probed with anti-peptidylarginine deiminase (*PAD*)2 antibody (**d**) or anti-PAD4 antibody (**e**). **f** Western blot of soluble fraction of ATRA/HL60 cells treated with indicated death-inducing stimuli and cultured in HBSS/fibrinogen/5 m M calcium for 24 hours and probed with anti-PAD2 antibody (**f**) or anti-PAD4 antibody (**g**). *PMA* phorbol 12-myristate 13-acetate, *IONO* Ionomycin, *GAPDH* glyceraldehyde-3-phosphate dehydrogenase

We reasoned that granulocytes undergoing cell death could release functional PAD enzymes into their local environment and that these could function to citrullinate extracellular fibrinogen. To test this hypothesis, ATRA/HL60 cells were cultured with fibrinogen while treated with stimuli that induce different types of cell death and fibrinogen citrullination in culture supernatants was assessed by western blot. ATRA/HL60 cells treated with Ionomycin, PMA or four cycles of freeze thaw (to induce primary necrosis) induced strong citrullination of fibrinogen while apoptotic ATRA/HL60 cells treated with UV did not. This finding persisted even after 24 hours in culture allowing for secondary necrosis and loss of membrane integrity of the UV-irradiated apoptotic cells (Fig. 3a). Blots were stripped and reprobed with anti-fibrinogen antibody to ensure that any negative signals were not due to degradation of fibrinogen itself by ATRA/HL60 proteases. The amount of total fibrinogen did not change in any culture group. Freeze thawed (necrotic) ATRA/HL60 lysates incubated for 24 hours without fibrinogen did not cause citrullination (Fig. 3b), indicating that the cells themselves are not the source of citrullinated fibrinogen.

To confirm that apoptotic cells do not lead to fibrinogen citrullination and that our observation was not unique to UV-irradiated cells only, we also tested whether ATRA/HL60 cells undergoing apoptosis secondary to staurosporine treatment [13] induced fibrinogen citrullination. Though staurosporine treatment induced apoptosis more slowly than UV irradiation, 37 % of cells were apoptotic at 4 hours and 76 % of cells were secondarily necrotic by 24 hours. Staurosporine treated ATRA/HL60 cells did not lead to citrullination of fibrinogen after 24 hours of culture (Fig. 3b). We also tested whether necrotic human blood-derived neutrophils citrullinated extracellular fibrinogen and found that they induced very rapid citrullination after only 5 minutes of incubation (Fig. 3c), and that freeze thawed neutrophils alone without incubation with supplemental fibrinogen did not contain citrullinated fibrinogen. Western blots of live unstimulated ATRA/HL60 cell pellets demonstrated binding by anti-PAD2 (Fig. 3d) and anti-PAD4 antibodies (Fig. 3e) and after incubating cell lysates for 24 hours, a broad range of bands between 76 and 24 kD were detected by anti-PAD2 antibody and a distinct band was detected at 31 kD by the anti-PAD4 antibody. It is likely that these additional bands represent PAD degradation products.

A potential interpretation of the finding that ATRA/HL60 cells citrullinate fibrinogen specifically when undergoing inflammatory but not apoptotic death is that apoptotic death effectively sequesters PAD enzymes and deactivates them before they can access the extracellular compartment to citrullinate other neighboring proteins even if allowed to undergo secondary necrosis. To directly test this hypothesis, we measured PAD2 and PAD4 in the supernatants of dying ATRA/HL60 cells. Supernatants of freeze thawed PMA- and Ionomycin-treated cells were bound by a monoclonal anti-PAD2 antibody at the PAD2 predicted molecular weight of 76 Kd, and additional bands, which likely represent degradation products. In contrast, supernatants of UV-irradiated cells were not bound with the anti-PAD2 antibody (Fig. 3f). Supernatants of freeze thawed cells demonstrated a faint band stained by a monoclonal antibody to PAD4 at 74 kD, the predicted molecular weight of PAD4, with a stronger band at 31 kD in the freeze thaw, PMA and Ionomycin groups, which is likely to be a PAD4 degradation product (Fig. 3g). Anti-PAD4 antibody did not bind supernatants of UV-irradiated cells. These results indicate that ATRA/HL60 cells undergoing inflammatory cell death, but not apoptotic death, release PAD2 and PAD4, and this correlates with citrullination of fibrinogen in the extracellular compartment.

## Discussion

Bone marrow precursors produce approximately 50 billion neutrophils per day. In order to maintain homeostasis, neutrophils undergo apoptosis spontaneously after 5 days in circulation and 1–2 days after trafficking to tissue. Inflammatory stimuli known to be risk factors for RA, such as periodontal infection or cigarette smoke, can lead to alternate types of neutrophil death including NETosis, autophagy and necrosis [14]. Dead or dying neutrophils can in turn directly alter immune responses positively or negatively depending on the inflammatory milieu, and the type of neutrophil death [15]. Modeling neutrophils using a differentiated promyelocytic leukemia cell line (HL60), we describe how inflammatory neutrophil death can lead to modification of extracellular proteins such as fibrinogen, which can become targets of ACPA.

As ATRA-differentiated cells express high levels of PAD2 and PAD4, it is reasonable to propose that dying cells could release these enzymes and that they could continue to be functional, particularly as the calcium levels in the extracellular space are higher relative to the intracellular compartment. A limitation of this work is that it is not possible to distinguish whether PAD2 or PAD4 was responsible for citrullination of fibrinogen in our assay, because they were both detectable in the cell media supernatants. It is worth noting that PAD4 was detected at a lower molecular weight than expected (31 kD) and this may have represented a non-functional degradation product, while PAD2 remained detectable at its predicted molecular weight of 76 kD. Additionally, it was not possible to induce NETosis without inducing concurrent necrosis and therefore, we cannot discern whether NETosis is required for extracellular citrullination. Freeze thaw, which induces only necrosis, was sufficient, however, to induce fibrinogen citrullination. Perhaps the more surprising finding is that apoptotic and even secondarily necrotic cells did not citrullinate extracellular fibrinogen in culture. This result indicates that death by apoptosis deactivates the PAD enzymes before they are released into the extracellular space and is consistent with prior work demonstrating that PAD4-mediated histone deimination is induced by inflammatory stimuli in neutrophils but not apoptosis [16]. Considering the vast numbers of neutrophils that undergo apoptosis daily, it stands to reason that this immunologically silent cell death would also have evolved mechanisms to limit modifications of neighboring proteins. Single nucleotide polymorphisms in PADI4 and PADI2 are associated with increased risk of RA. PADI4 risk alleles reside in the N-terminal domain, which is not the catalytic site of enzyme activity, and these risk alleles do not lead to enzymes that are hyperfunctional in vitro, leading to the hypothesis that the polymorphism may confer risk by increasing either protein or mRNA stability rather than increased protein function [11]. The work presented here supports the notion that increased PAD stability could contribute to RA risk by demonstrating that PAD enzymes released in the setting of certain types of cell death continue to function in the extracellular space.

While autoantibodies to citrullinated fibrinogen are highly specific to RA, citrullination of fibrinogen is not. Non-RA patient-derived atherosclerotic plaques contain both citrullinated fibrinogen and PAD4 [17]. As neutrophils express more PAD4 than any other hematopoietic cells, this result suggests that neutrophils may be responsible for fibrinogen citrullination in vivo. Further, neutrophil-derived serine proteases promote coagulation and thrombosis formation in vitro and in vivo [18] and known products of NETosis, such as DNA, MPO and citrullinated H3, can be detected in organized human thrombus [19]. The work presented here provides a direct link between the observations of the importance of neutrophils in thrombus organization and the observation that thrombus contains citrullinated fibrinogen.

## Conclusions

In conclusion, we found that granulocytes undergoing inflammatory but not apoptotic cell death lead to citrullination of the extracellular protein, fibrinogen. Fibrinogen citrullination correlates with detectable levels of PAD enzymes in the supernatant of granulocytes undergoing inflammatory but not apoptotic cell death. This work provides a direct link between previous observations on the role of neutrophils in thrombosis and citrullination of fibrinogen, and points yet another finger at neutrophils in the generation of post-translational modifications targeted by RA autoantibodies.

## Additional files

Additional file 1:
**Western blot of trans retinoic acid (ATRA)/HL60 at various time points probed with anti-citrullinated histone H3.** *NO STIM* no stimulation, *PMA* phorbol 12-myristate 13-acetate, *IONO* ionomycin. (PDF 199 kb) Additional file 2:
**FACS staining of trans retinoic acid (ATRA)/HL60 cells treated with staurosporine.** *T* time, *TOPRO* cell impermeant nucleic acid stain. (PDF 199 kb)

## Abbreviations

ACPA: anticitrullinated peptide antibodies (ACPA)
ATRA: trans retinoic acid
DAPI: 4,6-diamidino-2-phenylindole
DDT: DL-Dithiothreitol
FBS: fetal bovine serum
GAPDH: glyceraldehyde-3-phosphate dehydrogenase
kD: kiloDalton
MPO: myeloperoxidase
NETs: neutrophil extracellular traps
PAD: peptidylarginine deiminase
PBS: phosphate-buffered saline
PMA: phorbol 12-myristate 13-acetate
PMN: peripheral blood neutrophils
RA: rheumatoid arthritis
RPMI: Roswell Park Memorial Institute
