# Supplementary material for: Inflammatory but not apoptotic death of granulocytes citrullinates fibrinogen
Source: Arthritis Res Ther. 2015 Dec 17;17:369. doi: 10.1186/s13075-015-0890-0 (PMC4704541; doi:10.1186/s13075-015-0890-0)

Additional File 1: Western blot of ATRA/HL60 at various time points probed with anti-citrullinated histone H3

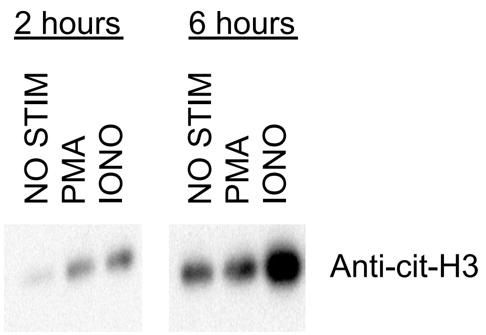

Supplement: Additional file 1: — Western blot of trans retinoic acid (ATRA)/HL60 at various time points probed with anti-citrullinated histone H3. NO STIM no stimulation, PMA phorbol 12-myristate 13-acetate, IONO ionomycin. (PDF 199 kb) [file 13075_2015_890_MOESM1_ESM.pdf]
