# Supplementary figures and images for: Inflammatory but not apoptotic death of granulocytes citrullinates fibrinogen
Source: Arthritis Res Ther. 2015 Dec 17;17:369. doi: 10.1186/s13075-015-0890-0 (PMC4704541; doi:10.1186/s13075-015-0890-0)

Additional File 2: FACS staining of ATRA/HL60 cells treated with staurosporine

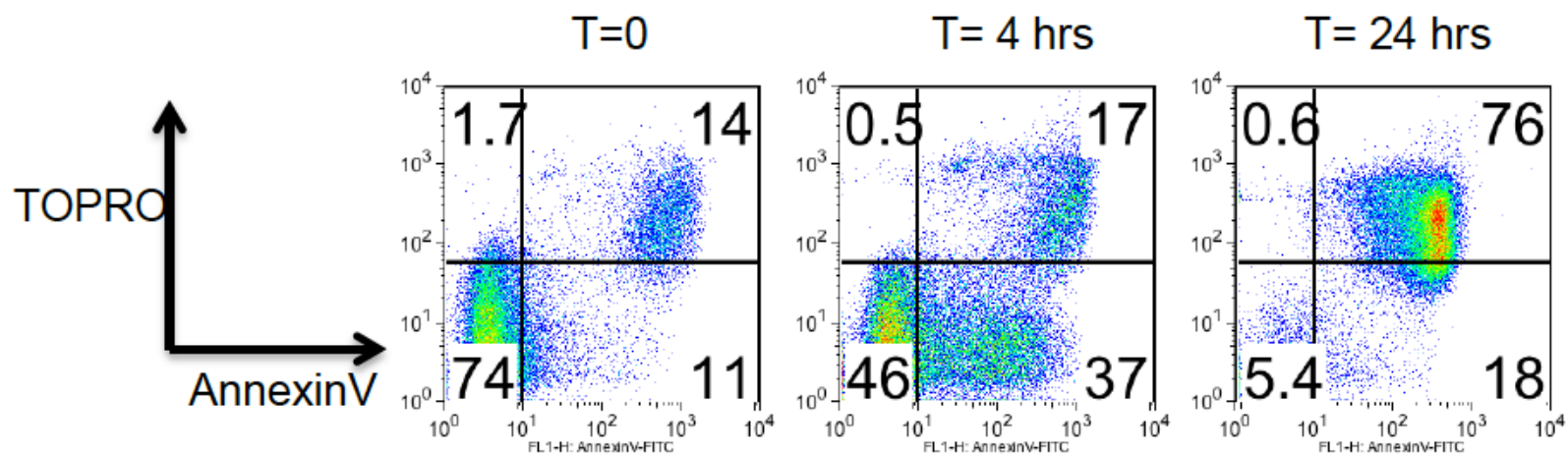

Supplement: Additional file 2: — FACS staining of trans retinoic acid (ATRA)/HL60 cells treated with staurosporine. T time, TOPRO cell impermeant nucleic acid stain. (PDF 199 kb) [file 13075_2015_890_MOESM2_ESM.pdf]
